# Supplementary material for: Theory of axo-axonic inhibition
Source: PLoS Comput Biol. 2025 Apr 21;21(4):e1013047. doi: 10.1371/journal.pcbi.1013047 (PMC12052214; doi:10.1371/journal.pcbi.1013047)
Supplement: Attachment — Submitted filename: Response to reviewers.pdf [file pcbi.1013047.s002.pdf]

Response to reviewers

Dear Editor and reviewers,

I am very glad that both reviewers appreciated this study, and many thanks for the detailed remarks. I have tried to address them as thoroughly as possible. The remarks of the reviewers have prompted me to look deeper into several interesting issues, and therefore the revised discussion is much extended. I have also added one panel (Fig. 3B), which shows that somatic inhibition does not raise the AP threshold, in contrast with axoaxonic inhibition, and one figure (Fig. 5), which shows that transient axoaxonic inhibition raises the threshold with essentially the same kinetics as the synaptic current.

While revising the manuscript, I also noticed a small error I made in the quantitative estimate of threshold change during theta rhythms (Discussion). I made a numerical error in the application of the formula. I also considered that the 20 ms decay time constant for the postsynaptic current measured by Tamás and Szabadics would probably be reduced to around 10 ms *in vivo*, but in fact that study was done at 36°C. As these errors go in opposite directions, the corrected estimate is not very different in the end.

I hope you will appreciate this revised version.

Best regards,

Romain Brette

Detailed response

Reviewer #1

This paper offers a tightly focused and important analysis examining the predictions of cable theory regarding GABAergic synaptic input on an axon initial segment. As described in the succinct but thorough introduction, the functional contributions of AIS input (particularly via chandelier cells), the effect of such synapses have been surprisingly unclear despite the intuition that they afford powerful inhibition. This work grounds this intuition in a straight-forward, clearly written, and effective analysis, showing that this can occur via an effective increase in firing threshold that could strongly suppress the activity of target pyramidal cells under biologically feasible conditions. This fills a key gap in the literature, and provides useful baseline predictions for the effect of chandelier cells on neuronal networks, even if the unique biophysics of the AIS might complicate the real biology.

> I thank the reviewer for this positive assessment.

Major Suggestions:

** One of the most striking examples of inhibitory specificity is that chandelier cells _only_ target the axon initial segments. Given that somatic inhibition can also strongly suppress the activity of target pyramidal cells, it would be useful to thoroughly and quantitatively compare the effect of perisomatic inhibition with that of axonal inhibition. This contrast is briefly mentioned to in the text around line 139/140, but an explicit comparison would be valuable in trying to distinguish the roles of these two non-overlapping categories of inhibition, and this paper is well-suited to bring both categories of inhibitory targeting into the same analytical framework.*

> I agree I could have made this more explicit. Because the soma is a current sink, perisomatic and axonal inhibition will produce the same effect at the soma. I added the following sentence (l. 140-141): "a given current will produce the same somatic depolarization or hyperpolarization, whether injected at the AIS or at the soma (Fig. 1B)".

The main difference is on the AP threshold, which is modulated by AIS inhibition but not by perisomatic inhibition, which is why I focused on that aspect. I now added a panel in Fig. 3 to make this clearer (Fig. 3B), where I show that the AP threshold does not change when the same inhibitory current is injected at the soma.

There is also another difference that I did not emphasize in the first version, which the second reviewer alluded to, namely on the time scale of inhibition. Perisomatic inhibition is filtered by the large somatodendritic capacitance, but the threshold modulation due to axoaxonic inhibition is not, because it acts through the axial resistance between soma and AIS. The effect is essentially instantaneous (more precisely, tracks the synaptic current). I added a figure with a simulation (Fig. 5) and corresponding section (Kinetics of threshold modulation, l. 224-242) to explain this effect.

I also added a section in the discussion to explain the differences between perisomatic and axoaxonic inhibition and to make some quantitative estimates (l. 279-318). Briefly, the effect of a synaptic current on the somatic potential, whether applied on the soma or AIS, is proportional to the input resistance R at the soma; the additional effect on AP threshold is proportional to Ra. Thus, the relative extra effect of axoaxonic inhibition is about Ra/R. But this is for a steady current; for fast currents, axoaxonic inhibition will be stronger as explained above.

** The biophysics of the AIS is complex (e.g. the very nice recent review by Jenkins and Bender, Axon Initial Segment Structure and Function in Health and Disease, 2025 Physiological Reviews), and there are structural hints that Chandelier cells react to this. In particular, chandelier synapses cluster onto cisternal organelles associated with ryanodine receptors suggesting some connection with calcium-dependent calcium release. There are also important differences in sodium and potassium channels compared to elsewhere on the neuronal surface. Notably, GABAergic input at the AIS seems focused on this biophysically distinct region and not just the initial span of AIS. The theory presented here is based in cable theory and offers a useful baseline model that has been generally lacking from the literature, but it would be useful to add some discussion of how AIS-specific biophysics might act on top of the phenomenon analyzed here.*

Very interesting point, thank you. I have added a section about this in the discussion (l. 393-418). Briefly, I see two important possibilities of interaction with the molecular organization of the AIS: 1) modulation of GABA receptors by calcium; 2) deinactivation of Cav3.2 channels of the AIS by hyperpolarization.

** There are some computational attempts to study AIS inhibition that are not discussed. Douglas and Martin 1990 (Control of Neuronal Output by Inhibition at the Axon Initial Segment) examined a similar geometry through simulation of Hodgkin-Huxley dynamics and came to a somewhat similar conclusion that AIS inhibition acts via controlling spike threshold. This study should be included in the otherwise excellent literature review in this paper, and it would be interesting to compare the results here with those found by Douglas and Martin. It would be instructive to compare the results here with those of this previous study, particularly as that study found it difficult for inhibition to overcome strong excitation, which is at least naively opposite to the estimates presented in the discussion (line 240–241). Similarly, Schneider-Mizell et al. 2021 included nonlinear biophysical simulations of inhibition at the AIS and compared to somatic inhibition, and could be included in this discussion.*

Indeed. I have added a section in the discussion (l.320-343). Unfortunately, neither of those two studies included the equations, parameters or code, so it is a bit difficult to assess precisely. Nonetheless, it seems clear that the study of Douglas & Martin did not correctly model the biophysics of the AIS (as probably most models at that time). Note that they found an effect on the rheobase, i.e., current threshold, and not on the voltage threshold (which was not measured). Any synaptic current at the soma would change the rheobase by an amount equal to the opposite to that current, and this is true for somatic inhibition as well as axoaxonic inhibition (because the soma is a current sink). What is special with axoaxonic inhibition is that it also modulates the voltage threshold, but this was not addressed (and probably did not occur, given what I understand of their model).

The model of Schneider-Mizell et al. might suffer from the same problem because it is based on blind optimization using somatic recordings, but it is hard to say as the optimized parameter values are not reported. Note also that in that study, the AIS geometry and position were in fact not measured, but defined as the proximal region of the axon from soma to 37 µm away.

Minor points:

** Line 96: A citation for the axial resistance of layer 5 pyramidal cells would be good.*

This is more of an estimate. I added some details in the methods. A theoretical estimate based on AIS diameter of 1-1.5 µm and intracellular resistivity of 100-150 Ω.cm gives 0.57-1.91 MΩ/µm. We can also estimate it from the patch clamp data of Hu & Bean (2018), who did simultaneous patch clamp measurements in the soma and axonal bleb. I find 0.8 $\pm$ 0.6 MΩ/µm. Thus, there is quite some uncertainty, but the order of magnitude is about 1 MΩ/µm.

Reviewer #2

This is an excellent paper. Dr. Brette presents an analysis of the effect of GABAergic synaptic currents occurring at the axon initial segment, as occurs with axon-axonic synapses. The analysis is presented in a highly accessible manner that nicely communicates the concepts as well as the results to a general reader. The presentation nicely combines a simple conceptual view of voltage drops across the axon coupling the AIS to the soma with full simulations of neuronal firing. The paper is exceptionally well-written. It is concise and clear in both the presentation of results and in the discussion.

I thank the reviewer for this positive assessment.

I have only two suggestions for the author to consider.

One is an explicit statement about how the effect of a given synaptic conductance at the AIS would differ from the same conductance on the soma. This is addressed indirectly in lines 185-194 in the context of analyzing synapses position along the AIS but a sentence explicitly comparing to a soma location might be useful.

Absolutely. The first reviewer also made the same suggestion. Therefore, I added a section on this in the discussion (l. 279-318). Briefly, a current on the proximal axon flows towards the soma and therefore has the same effect at the soma as a somatic current, but it additionally changes the AP threshold. I added a panel (Fig. 3B) to show the difference more explicitly. In the discussion, I make some quantitative estimates.

Another difference is related to your second remark below.

The second is a more complete consideration of the transient nature of the GABAergic currents. The analysis is of the steady-state situation but this is a simplification since the time-course of the synaptic conductance from a single firing of a presynaptic GABAergic is not long relative to the time course of capacitative charging. This point is alluded to in lines 236-240 but perhaps a more explicit statement would be helpful to the reader.

I would leave both of these suggestions to be considered or rejected as the author feels is best.

This is a very relevant point. In fact, a very peculiar aspect of axoaxonic inhibition is that its effect on threshold is essentially instantaneous (i.e., tracks the synaptic current). This is because the voltage gradient between soma and axonal injection follows Ohm's law (because the relevant resistance R_a is through the cytosol, not through the membrane). I added a figure (Fig. 5) that shows this more explicitly, as well as a discussion paragraph.
